# A Learning Theory Approach to Attachment Theory: Exploring Clinical Applications

**DOI:** 10.1007/s10567-021-00377-x

**Published:** 2022-01-30

**Authors:** Guy Bosmans, Leen Van Vlierberghe, Marian J. Bakermans-Kranenburg, Roger Kobak, Dirk Hermans, Marinus H. van IJzendoorn

**Affiliations:** 1 Clinical Psychology, KU Leuven, Leuven, Belgium; 2 Clinical Child and Family Studies, Vrije Universiteit Amsterdam, Amsterdam, the Netherlands; 3 Department of Psychological and Brain Sciences, University of Delaware, Newark, USA; 4 Centre for Psychology of Learning and Experimental Psychopathology, KU Leuven, Leuven, Belgium; 5 Department of Psychology, Education and Child Studies, Erasmus University Rotterdam, Rotterdam, the Netherlands; 6 Research Department of Clinical, Education and Health Psychology, Faculty of Brain Sciences, UCL, London, UK

**Keywords:** Attachment, Intervention, Learning theory, Early childhood, Middle childhood, VIPP-SD, Attachment-based Family Therapy

## Abstract

Although clinicians typically acknowledge the importance of insecure attachment as one factor that can contribute to children’s psychopathology, translating attachment theory into clinical practice has proved a challenge. By specifying some of the mechanisms through which the child’s attachment develops and changes, learning theory can enhance attachment based approaches to therapy. Specifically, interventions building on operant (parent management training) and classical (exposure therapy) learning can be used to stimulate new learning that increases the child’s security and confidence in the parent’s availability and responsiveness. To explore the clinical application and utility of a Learning Theory of Attachment (LTA), we focus on two attachment-focused interventions: Video-feedback Intervention to promote Positive Parenting and Sensitive Discipline (VIPP-SD) and Middle Childhood Attachment-based Family Therapy (MCAT). VIPP-SD is an evidence-based parent management training designed to promote sensitive parenting and secure attachment in early childhood. MCAT is a recently developed intervention that uses exposure to stimulate secure attachment in middle childhood. LTA sheds light on the mechanisms set in train by VIPP-SD and MCAT facilitating the induction of professionals in clinical applications.

**Supplementary Information:**

The online version contains supplementary material available at 10.1007/s10567-021-00377-x.

In the current contribution, we review how the basic principles of our Learning Theory of Attachment (LTA) might inform and be applied to clinical practice. We demonstrate that LTA can help understanding how early attachment relationships become incorporated in children’s minds. Beginning in infancy, children experience major developmental shifts in how they maintain attachments to their caregivers that may contribute to fluctuations in their attachment security (Groh et al., 2014). The LTA identifies opportunities to stimulate shifts toward more secure attachment as children grow older. Therefore, we will illustrate the clinical utility of the LTA by considering two interventions that were designed to stimulate and/or repair secure attachment bonds in young children and in middle childhood (the age period that typically starts when children are 6–7 years old and ends around 12–13 years). Understanding this process will illuminate what goes on in attachment and parenting interventions and may also help us sharpen our interventions to make them more effective. To set the foundation of this argument, we first review the basic premises of attachment and then present and discuss LTA. Finally, we will examine how LTA shows up in the two attachment-focused therapy models. It should be emphasized that the LTA itself is not a therapy or intervention but a theory that seeks to elucidate the mechanisms that may be targeted by clinicians and other professionals to increase children’s attachment security.

Enhancing children’s attachment security, that is their confidence in a parent’s or other caregiver’s ability to provide protection and care when needed (Bowlby, 1969), might add an important component to the effectiveness of parenting programs and evidence-based psychotherapies for children (Bosmans, 2016). Unfortunately, there are few manualized and empirically supported interventions to increase children’s trust in their parents or caregivers. Designing such interventions has proved challenging given attachment researchers’ struggles with clearly specifying and measuring the processes through which children develop and maintain secure attachment relationships (e.g., Verhage et al., 2016). Bosmans et al. (2020) began to address this problem by proposing that attachment development can at least in part be explained with principles of safety learning. This is a specific type of conditioning, also called conditioned inhibition, whereby stimuli become predictors of relief from stress after repeated learning trials during which these stimuli precede the experience of relief (Craske et al., 2018). Applied to attachment theory, Bosmans et al. (2020) argued that children’s trust in parents reflects in part a conditioned level of certainty or confidence that parents will provide support when the child encounters distressing situations.

## Traditional Attachment Theory

According to attachment theory with its Darwinian roots, every newborn infant is preadapted to develop an attachment relationship with an attachment figure (Bowlby, 1969, 1988). Newborns cannot survive without the protection, stress- and thermoregulation and nurturing by caregivers whose ‘inclusive fitness’ through transfer of their genes into next generations depends on the survival of offspring into procreative age. On this species-wide foundation of preparedness to become attached, individual differences in type of attachment relationships emerge during the first few years of life. When children experience that attachment figures consistently provide sensitive support to (di)stress, they tend to become securely attached (Bowlby, 1969). These experiences are stored in what Bowlby (1969) labeled Internal Working Models (IWM) to emphasize their dynamic nature, and their core of cognitive representations of past experiences reflecting the degree of confidence in future support from significant others (Bretherton et al., 2005). Securely attached children are suggested to strike a balance between exploring their environment and seeking proximity to and support from attachment figures during (di)stress (Dujardin et al., 2016). Attachment figures can be any caregiver with whom the child interacts on a regular basis and who might provide protection, nurturance and emotional support (Bakermans-Kranenburg, 2021). In this contribution, we will focus on parents as primary attachment figures. However, what follows can be applied to any attachment figure.

When children experience parents to be less consistently available for support or consistently unavailable, they may develop an insecure attachment relationship with them and an insecure IWM about their parents’ availability. On the one hand, children may become more ambivalent in their attachment relationships and keep their attention focused on the attachment figure instead of balancing it with exploring the wider social or physical environment. This means that their attention and behavior express anxiety for rejection, and that they continue to seek support even in the absence of distress (Cassidy, 2016; Kobak et al., 1993). On the other hand, children may develop more avoidant behavior in an attachment relationship with parents who are consistently ignoring or even rejecting the child’s bids for care and protection in times of distress. Insecure-avoidantly attached children try to keep their attention focused on the environment, away from the attachment figure. They minimize the overt signaling of attachment needs during distress presumably in an attempt to avoid potential parental rejection and to keep optimal proximity to a less than optimally protective caregiver (Cassidy, 2016; Kobak et al., 1993).

Accumulating research and meta-analyses robustly show that insecure attachment predicts higher levels of internalizing and externalizing problems across the lifespan (e.g., Groh et al., 2017). The effect sizes suggest that insecure attachment is not psychopathology itself. Instead, research shows that insecure attachment provides a context in which risk factors develop that are linked to elevated chances of developing psychopathology. These include, among others, less adequate emotion regulation (Verhees et al., 2021), social skills (Bastin et al., 2021), and maladaptive cognitive schemas about the self and others (Simard et al., 2011). As a result, children may divert their attention away from their caregivers and wait longer to seek their support during distress, or they keep focused on the attachment figure at the cost of exploring the environment, both attentional strategies resulting in higher chances of developing psychopathology (e.g., Dujardin et al., 2016). Insecure attachment has been proposed as a robust, transdiagnostic risk factor, rendering it a valuable target for therapy or intervention.

It should be noted that disorganized attachment (Main & Solomon, 1990) as a hypothesized consequence of experiences of traumatizing fear will be left out of our discussion of attachment implications for therapy and interventions. At this point in time still too many unsettled theoretical and empirical questions limit the possibility of deriving specific clinical implications for disorganized attachment (e.g., see Granqvist et al., 2017).

## A Learning Theory of Attachment

Attachment theory has often been often criticized for being vague about the concept of the IWM and on how experiences with sensitive and supportive parents translate to its development (e.g., Rutter, 2014; Thompson, 2016). Indeed the IWM seems to be the black box in attachment theory (Bosmans et al., 2020) illustrating how hard it is to identify the mechanisms of attachment development on the micro level of dyadic relationships. Knowledge about these mechanisms might be helpful to identify the intervention targets that are needed to stimulate or restore secure attachment development (Bosmans, 2016). In this section, we briefly present the recently proposed LTA (see Bosmans et al., 2020, for details). This theory describes mechanisms of attachment development and how experiences with parental support or lack of support are internalized into internal working models.

### Safety Learning and Secure Base Script Development

Although Bowlby left room for learning processes in attachment development, attachment researchers were often opposed to integrating learning and attachment theories (Bosmans et al., 2020). However, empirical data provide some evidence that variation in attachment (in)security may at least partly be explained by learning processes that play a role in safety conditioning. This does not contradict the observations leading attachment researchers to emphasize the inborn nature of the species-specific preparedness to become attached to a protective caregiver (e.g., Kraemer, 1992; Rajecki et al., 1978). According to the LTA, infants are biologically prepared to be attuned to their caregivers as stimuli that facilitate attachment-related learning through classical and operant conditioning (Bosmans et al., 2020).

**Fig. 1:**
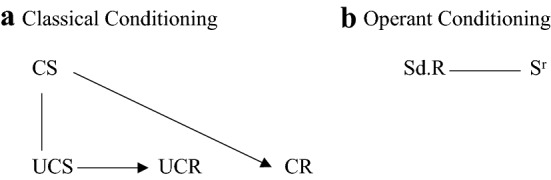
Classical and operant conditioning. **a** depicts that if the Conditional Stimulus (CS) gets paired with the Unconditional Stimulus (UCS), which automatically elicits the Unconditional Response (UCR), the CS elicits a Conditional Response (CR). **b** Depicts that a discriminative Stimulus (Sd) elicits a behavioral Response (R) if that behavior is reinforced by increase of positive consequences or decrease of negative consequences (reinforcing Stimulus, Sr). Figures 2 and 3 will illustrate the application of these schemas to attachment-related constructs

In brief, classical conditioning refers to learning about the co-occurrence of stimuli/events, while operant conditioning refers to how specific behaviors are related to their antecedents and consequences. Classical conditioning (see Fig. 1) occurs when an initially neutral stimulus (the Conditional Stimulus; CS) is presented in association with a meaningful stimulus (typically food or a pain-inducing stimulus like an electro-shock; this is named the Unconditional Stimulus; UCS). The UCS gets its meaning because it is by default linked to several positive or negative reactions (e.g., reduction of hunger or increase of stress, the Unconditional Reaction; UCR). When the CS and UCS are repeatedly presented together, the CS becomes the predictor of the UCS/UCR and elicits a Conditional Response (CR). Once the CS elicits a CR, conditioning is said to have occurred. For example, the CS can elicit the expectation (CR) that the rewarding or unpleasant experience of a parent’s (un-)availability during distress will follow.

The CR occurs at different levels of processing, ranging from more strategic to more automatic processes (Gawronski & Creighton, 2013). An example of a more strategic CR is the expectation about parent’s availability that one can report about. When these expectations reflect specific themes, they are called cognitive schemas, and when the expectations reflect a chain of events that might unfold, they are called cognitive scripts. Examples of a more automatic kind are biases in the cognitive processing of the CS such that information about the CS is more easily recalled, attentionally encoded, or interpreted in congruence with learned expectations about the CS. Such information processing biases are less easy to control. They may explain why certain expectations about the CS cannot be changed by merely exposing individuals to corrective information about the CS (Baert et al., 2011).

Operant conditioning (see Fig. 1) occurs upon exposure to a Discriminant Stimulus (S^d^). This is a context in which a certain behavior (R) is likely to be reinforced (Reinforcing Stimulus; S^r^). Behavior is repeated when followed by a positive reinforcer (e.g., feeling cared for, annotated as +Sr+) or the omission of negative reinforcers (e.g., reduction of stress, annotated as −Sr− or °Sr−). However, when behavior is followed by negative reinforcers (e.g., feeling rejected, annotated as +Sr−) or by the reduction of positive reinforcers (e.g., absence of care, −Sr+or °Sr+), behavior fades out. For an overview of all possible reinforcers, see Table 1.

**Table 1:** Reinforcers in operant conditioning

Procedure	Type of reinforcer	
	Positive (+)	Negative (−)
Appears (+)	+Sr+	+Sr−
Disappears (−)	−Sr+	−Sr−
Does not appear (°)	°Sr+	°Sr−

Sr: Reinforcing Stimulus; Behavior increases when followed by +Sr+ because a pleasant outcome is obtained and when followed by −Sr− or °Sr− because it avoids unpleasant outcomes. Behavior decreases when followed by −Sr+ or °Sr+ because anticipated positive outcomes are not obtained and by +Sr− because the behavior elicits negative outcomes

**Fig. 2:**
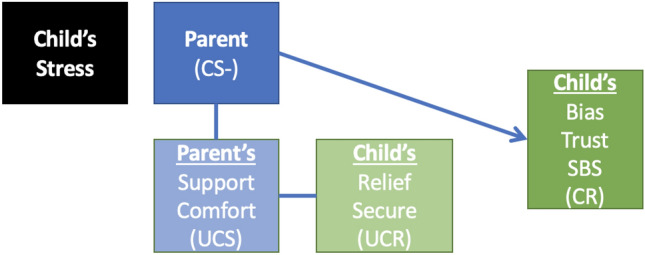
Safety conditioning and the learning theory of attachment. *CS*− Conditional Stimulus predicting that the negative effects of stress will stop; *UCS* Unconditional Stimulus; *UCR* Unconditional Reaction; *CR* Conditional Reaction; *SBS* Secure Base Script

Safety learning is a specific type of conditioning that occurs when individuals are exposed to distressing stimuli and when these stimuli are presented together with a CS which predicts the omission of the feared outcome (indicated as CS−). Specifically, this occurs when an organism learns that a stimulus (CS1) is followed by an aversive consequence (CS1+). However, when CS1 is presented in compound with another stimulus (CS2), the aversive consequence does not occur (CS1/CS2−). Thus, the CS2 becomes a conditioned inhibitor or a safety signal. In the LTA, the CS− is typically the parent or whoever is the child’s attachment figure. Through repeated learning experiences, the CS− will elicit confident expectations that negative emotions associated with the distressing stimulus can be effectively managed. According to the LTA, each interaction with the parent when the child is distressed can be considered a single learning trial (see Fig. 2). When the parent provides support (the UCS), children will automatically experience decreased distress (e.g., relief) and increased positive feelings of being cared for and reassured of their parents’ availability and responsiveness (Bosmans et al., 2020). The reduction of distress combined with positive feelings of security can be considered the UCR. Through a process of classical conditioning, over learning trials, the parent becomes a safety cue (CS−) that, for the child, predicts support, comfort and relief (UCS/UCR) during distress due to which children develop trust in the availability and support of the parent (CR).

After repeated learning trials, securely attached childrens’ confident expectancies in their parents’ availability and support become organized in a secure base script to be considered part of a secure IWM of attachment (Waters & Waters, 2006). A secure base script reflects a child’s knowledge of the interactions during which the child experiences that parents successfully provide support during distress (also described as *secure base interactions*)*.* Because these interactions always follow the same scenario, the secure base script refers to the expectation that upon exposure to distress, care-related interactions will follow this predictable scenario. Specifically, when children encounter a threat or challenge, distress is communicated to the parent directly (the child shows distress, turns toward the parent and signals the need for comfort). The parent reads the child’s behavior and understands that the child needs comfort and support. Subsequently, the parent responds with emotional support and may provide practical support. This open communication and the parental responses to this communication help the child get back on track and return attention to exploring and engaging the environment.

**Fig. 3:**
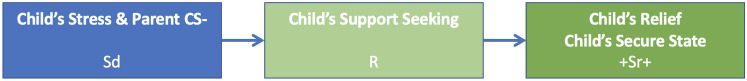
Operant conditioning and the Learning Theory of Attachment. *Sd* Discriminative Stimulus; *R* behavior; +Sr+ : a positive reinforcing stimulus follows

A secure or confident expectation that the caregiver will be available and responsive increases the likelihood that the securely attached child will enact the secure base script when confronted with dangerous, distressing, or challenging situations. When support seeking behaviors are met with sensitive parental response, these behaviors are further reinforced through a process of operant conditioning (see Fig. 3; Bosmans et al., 2020). Specifically, when secure children encounter distressing situations (Sd), they will seek support (the behavior, R), which is reinforced by the reduction and regulation of distress and the comfort provided by the parents’ sensitive response (Sr). Support seeking behavior will be repeated when followed by feeling cared for (+Sr+) or the reduction or avoidance of stress (−Sr− or °Sr−). However, when support seeking is followed by feeling rejected (+Sr−) or by interrupted care or absence of care (−Sr+ or °Sr+), support seeking behavior will fade out.

### Insecure Attachment Learning and the Development of Negative Interpersonal Schemas

In contrast to the securely attached child who likely experiences sensitive and responsive support (UCS) from the parent (CS) during distress, the insecurely attached child experiences the parent as unavailable or unresponsive (UCS) when the child encounter distressing situations which elicits painful feelings of being rejected or ignored (UCRs). As a result of repeated experiences with an insensitive caregiver, the child not only anticipates the parent’s failure to provide safety and protection, but also anticipates painful feelings of rejection (CR). Instead of developing a secure base script of how to signal and rely on others at times of distress, insecure children develop cognitive schemas about others as being unavailable (CR) and about themself as being unworthy or incompetent (Simard et al., 2011). Examples are Young’s early maladaptive schema’s (Young et al., 2003; see Supplementary File 1) that are adaptive to the (less than optimal) environment in which they were developed (Hochberg & Belsky, 2013) but put individuals at elevated risk to develop relational problems and even psychopathology at a later stage. Main (1990) already argued that insecure attachment behavioral strategies could be adaptive as a short-term strategy for reducing conflict with the caregiver, and Belsky and others suggested that these strategies may be embedded in fast reproductive strategies (Hochberg & Belsky, 2013).

Maximizing the signaling of attachment needs may have the immediate advantage to increase the likelihood of support from a less available parent (+Sr+). However, because the parents is inconsistently available, the +Sr+ will only intermittently follow support seeking behavior. Through this intermittent reinforcement behavior increases in frequency and becomes highly resilient to extinction. At the same time, it increases the risk to elicit negative reactions from the caregiver and triggers rejection (+Sr−), in which case the child again feels the frustration of not being cared for (+Sr−). If support seeking is only intermittently reinforced, it becomes a highly stable behavioral pattern (Bosmans et al., 2020). In the long run, repeated experiences of disappointment culminate to frustration and anger (+Sr−) that feeds into demanding and hostile behavior that further distorts signaling of attachment needs and that perpetuates an insecure cycle of interactions with significant others (Kobak & Bosmans, 2019). Minimizing the signaling of attachment needs has the immediate advantage that it helps avoiding feared feelings of rejection (°Sr−). Moreover, resolving distress quasi-autonomously gives a sense of competence (+Sr+). However, in the long run, these strategies may come with a sense of isolation and enhanced chronic stress when they are insufficient to meet the need for proximity and protection (+Sr−).

## Exploring the Clinical Applications of the LTA

The LTA proposes that to understand attachment development, it is important to distinguish trait- and state-like attachment components that interact over time and may explain changes at the trait attachment level brought about by therapy or interventions. State attachment refers to the sense of being loved and comforted during distress or lack thereof and of the associated sense of relief or rejection (Gillath et al., 2009). Specifically, state attachment can be considered equivalent to the UCR. Hence, state attachment is dynamic and responsive to fluctuations in interactions with caregivers. If the CS_parent_ − UCS_support_ contingency increases, children will more frequently experience state secure attachment and high levels of state trust. When the CS_parent_ − UCS_support_ contingency decreases, children will more frequently experience state insecure attachment. State attachment is not only affected by ongoing care-related interactions with parents (Vandevivere et al., 2018), but also by cues that activate memories of past experiences of support or lack thereof (Bosmans et al., 2014). Over learning trials, more frequent experiences of state (in)secure attachment will eventually be reflected at the trait attachment level. Trait attachment can be situated more at the level of the CR and refers to a generalized expectation that the caregiver is mostly available and responsive for support during distress or mostly unavailable and unresponsive. This generalized expectancy feeds into the child’s attachment-related information processing biases, explicit and more automatic attachment-related expectations, and secure base script or alternative insecure scripts and early relational schema development.

CRs change when the CS–UCS contingency changes over time. New learning subsequently inhibits the activation of old CRs and old behavior extinguishes (Craske et al., 2018). Consequently, restoring or promoting secure attachment in children, adolescents and adults (‘from cradle to grave’, Bowlby, 1969) is feasible and a relevant target for clinical intervention. Although this is a hopeful message for clinicians, there are two important limitations to take into account. First, old knowledge never gets unlearned (Bouton et al., 2021; Rescorla & Wagner, 1972). So, it is always possible that old CRs to CSs get reactivated. If the CS–UCS contingency gets reversed again, or if old memories get primed by contextual cues, old CRs and related behaviors are likely to re-emerge. Because CS–UCS contingency is never 100% or 0%, each individual is likely to be confronted with a composite of positive and negative attachment experiences, and even though these memories might not immediately manifest at trait attachment level they can still be reactivated and influence the cognitive processing of ongoing interactions and influence subsequent interpersonal behavior. Second, attachment (in)security becomes more resistant to change with increasing age (Waters et al., 2020). Experiences get increasingly stored as prototypes that form a blueprint, negatively affecting the likelihood that new information updates expectations (Waters, Facompre, et al., 2019; Waters, Facompré, et al., 2019; Waters et al., in press). This implies that restoring or stimulating secure attachment development will be easier at younger ages.

In essence, the LTA suggests that attachment-focused interventions should aim at creating corrective learning experiences during which parents provide care when the child experiences distress. To alter the CS–UCS contingency, multiple learning trials will be needed during which the quality of the parent–child interactions is improved. For younger children it is likely sufficient to merely train parents to respond in a more supportive way to their child during distress. The LTA predicts that this will increase the CS_parent_ − UCS_support_ contingency, thus increasing children’s trait attachment security. Older children have developed more explicit insecure schemas (Rijkeboer & de Boo, 2010), with increasingly stronger information processing biases (e.g., Dudeney et al., 2015). Because such biases reduce the likelihood that children will notice and interpret parents as being more supportive than expected (Bosmans, Sanchez-Lopez, et al., 2019; Bosmans, Waters, et al., 2019), merely exposing children to corrective information during care-related interactions with parents will not result in corrective learning experiences (Baert et al., 2011). One way to overcome this, is to make the corrective experience emotionally more salient. This can be done by explicitly activating negative expectations during care-related interactions. If negative expectations and fear are more strongly activated, rule-violations get better encoded and more likely result in changed expectations and new behavioral strategies (Craske et al., 2014).

### The Insecure Cycle: A Model to Capture the Familial Dynamics Underlying Insecure Attachment Development

**Fig. 4:**
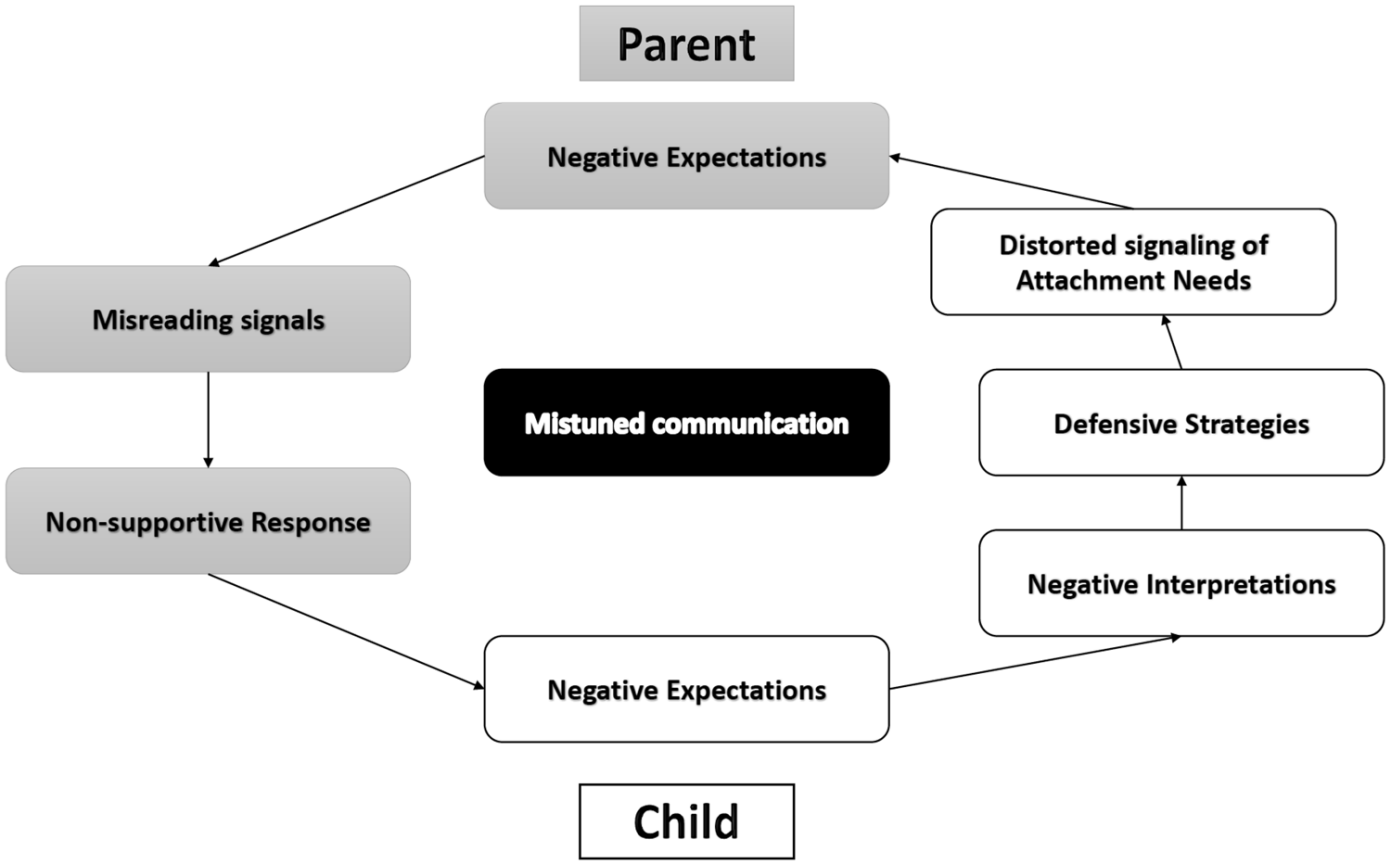
The Insecure Cycle of mistuned communication between parent and child. This figure shows how mistuned communication between children and parents sets off a negative interaction that feeds into the development of insecure attachment-related expectations, anxious and avoidant defensive strategies that lead to children’s distorted signaling of attachment needs to which parents respond in a non-supportive way that further confirms children’s insecure attachment expectations

In order to make clear how family dynamics leading to insecure attachments look like from a LTA perspective we outline its application to the development of the child-parent relationship which might result in self-perpetuating cycles to be dealt with in interventions. The model of the insecure cycle (Kobak & Bosmans, 2019; see Fig. 4) helps capturing the family dynamics that explain how the interactions between children and parents (CS) unfold up to the point that children no longer experience their parents as support figures (UCS-UCR). The insecure cycle model builds on the basic assumption that infants’ inborn tendency to form attachments continues into later developmental periods as a need to rely on parents in difficult situations (*need for care)* and in parents’ need to protect and encourage their children’s competence and well being (*need to care*). Successful pursuit of the child’s attachment needs and the parent’s caregiving needs is repeatedly threatened by cycles of mistuned communication. Mistuned communication can prime children’s negative memories of past interactions with parents that were experienced as unsupportive. Mistuned communication can reinforce children’s negative expectancies of parents availability and responsiveness. The child’s fear that parents will be unavailable in situations of danger or distress activates defensive attentional and behavioral strategies aimed at reducing distress. These affect regulation strategies either reflect amplified approach motivated by the hope of receiving care, or more common shifts of attention away from attachment figures motivated by a search for independence and control (Kobak et al., 1993; Main, 1990). These two defensive strategies translate into behavior that either exaggerates the display of attachment needs as clingy or demanding and frustrated, or that seems to minimize needs through quasi-autonomous behaviors.

Children’s insecure attachment behaviors run the risk of perpetuating insecure cycles instead of triggering protective parenting. This is because the child’s attachment signals challenge caregivers’ ability to read and respond to their child’s needs for protection and support. The child’s angry, demanding or withdrawn behaviors may prime parents’ own past negative attachment memories or they may reinforce parents’ negative view of their child, their child’s development, and of themselves as failing in their parenting task or as not being worthy to be loved. These negative expectations bias parents toward reading the child’s signals as inappropriate behaviors that need to be stopped. As a result, they miss the child’s underlying need for protection and support. Instead, they unintentionally confirm the child’s lack of trust in their parents’ availability and responsive support. This cycle of children’s insecure signals and of parents’ misattuned responses might reduce the CS_parent_ − UCS_support_ contingency and promote children’s trait-like expectations that parents will not provide care during distress.

The insecure cycle can also be intensified by well-meant advice from therapists and parent management trainers to parents to change their approach to their children. Such advice can activate parents’ own memories and representations of past experiences with their attachment figures during which they felt inadequate, insufficient, a failure, or not loved. This can activate anxious or avoidant defensive strategies in the parents’ approach to the therapist and can set off an insecure cycle between the therapist and the parents. This may decrease therapy alliance and increase drop-out risk. Hence, working with families’ insecure cycles requires therapists to acknowledge the parents’ competence (they know their child best) and their desire to be a good parent. Therapists may prevent getting drawn too deeply into insecure cycles by always keeping their focus on the parents’ underlying attachment needs and related fears. This allows therapists to become an ad hoc attachment figure fostering parents’ positive or corrective attachment learning experiences (Bowlby, 2005; Byng-Hall, 1990; Verschueren & Koomen, 2012).

Finally, it is important to note that the insecure cycle model complements the coercive cycle model of parent–child interactions by adding an affective layer to the reinforcers that explain the maintenance of children’s challenging behaviors. A coercive cycle is an interactional pattern that is typically observed in children with behavior problems (Reid et al., 2002). The coercive cycle has been described as an operant conditioning process during which children show increasingly demanding behavior to achieve their immediate goals (e.g., receiving toys or candy) and during which parents increasingly concede to the child’s demands to escape from the conflict and stress raised by the child (see left half of Table 2). However, these reinforcers disregard possible underlying attachment-related relational meanings or functions. The right half of Table 2 suggests some possible insecure cycle-related reinforcers that can perpetuate the coercive cycle and that can increase the risk that coercive cycles will repeat in the future.

**Table 2:**
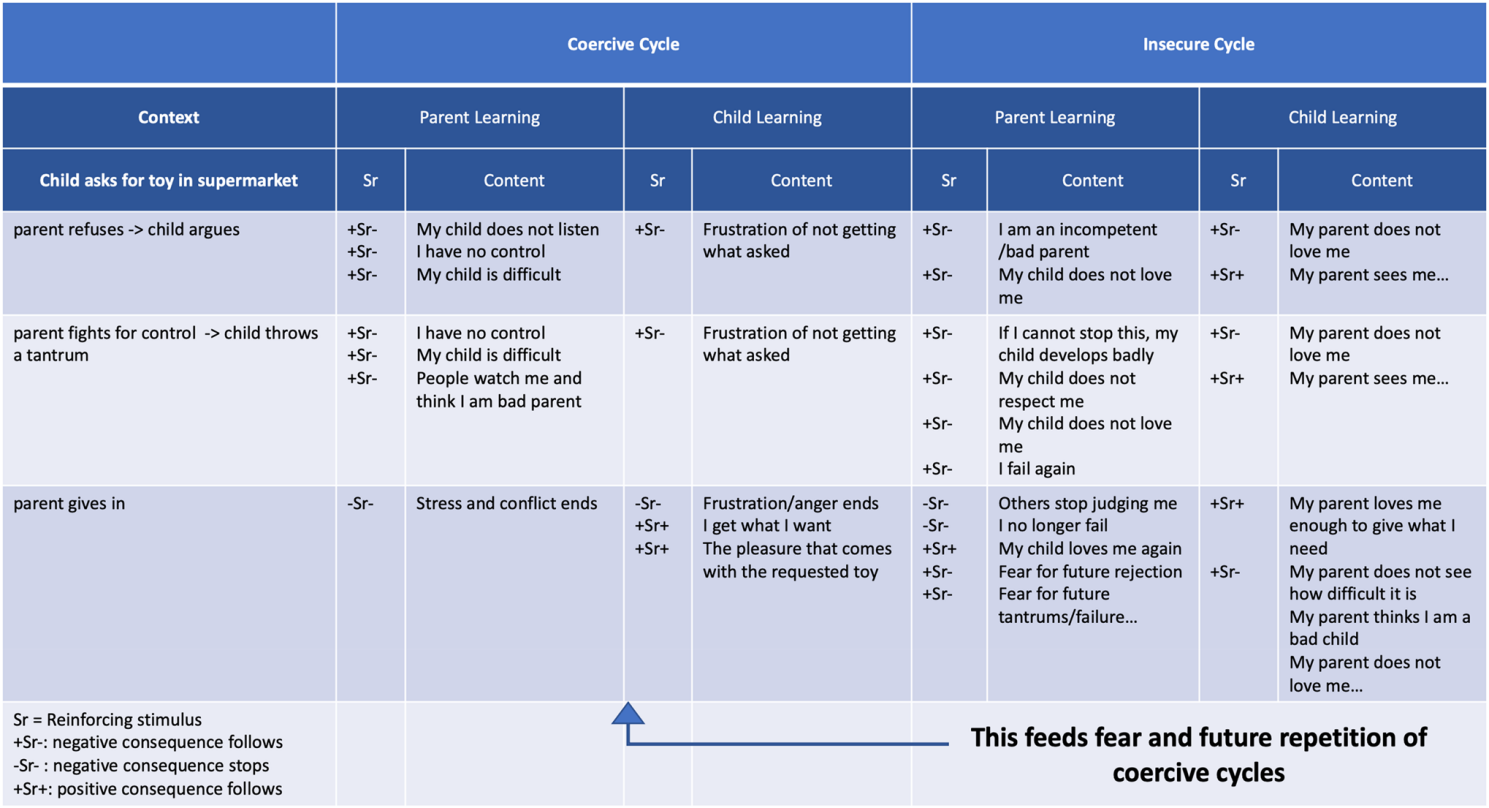
Integrating the coercive and insecure cycle

When parents feel that their child refuses to obey and that they fight in vein to control their child, a set of negative reinforcers can be elicited. Parents can feel incompetent or loved nor respected by their child (+Sr−). Subsequently giving in to the child’s demands is positively reinforced by decrease of distress linked to failure and rejection (−Sr−) and return of a positive interaction with the child who shows love and connection (+Sr+). When children feel intense desire for an object and parents refuse to give in, for children this can feel as a lack of acknowledgement of their stress and as rejecting (+Sr−). In addition, for some children who feel not seen by their parents, parents’ negative attention can feel rewarding as well (+Sr+). To reach their desired goal, children will increase their demanding behavior until the parents finally give in (+Sr+).

However, when parents give in after a conflict with the child, this typically comes with more or less explicit emotionally laden negative relational messages, expressing irritation and criticism towards the child and explicit or implicit rejection of the child (e.g., McCarty et al., 2004). Hence, although at first sight the fact that children get what they want seems like a mere positive reinforcement of their unwanted behavior, these parental behaviors are a discriminative stimulus for children (Sd) which activates insecure schemas and fears in children about not being loved and being a failure in the eyes of their parents. This is actually a CR that reflects children’s past classical learning experiences during which their parents (CS_parent_) got paired with negative care-related experiences (UCS_rejection_). These fears elicit more distorted signaling of attachment needs (e.g., oppositional behavior, R_child_). This behavior gets reinforced because it shifts the focus away from feelings of loneliness and abandonment (−Sr−), because it prevents those feelings to be activated (°Sr−), or because it elicits parental attention (+Sr+) and the hope that parents might respond more sensitively after this new attempt (+Sr+). However, this in turn becomes a Sd for the parents due to which the insecure cycle continues. We propose that the reinforcers identified in the coercive cycle (reduction of stress in the parent, achieving desired goals in the child) have a stronger immediate impact than the reinforcers identified in the insecure cycle. The latter, however, shape the relational environment for coercive cycles to reoccur as the resulting negative appraisals of each other increase the likelihood for new coercive cycles to set off.

The model of the insecure cycle integrates family dynamics and learning processes. When families get caught up in insecure cycles, CS_parent_ − UCS_support_ contingency decreases and children learn that they cannot trust in their parents’ availability and support. Hence, to stimulate or restore attachment development, interventions are needed that prevent, interrupt, and/or restructure insecure cycles. This can be done by altering the context (Sd-oriented interventions), by altering the consequences of parent and child behaviors (Sr-oriented interventions), and by altering the meaning that children attribute to parents and that parents attribute to children (CS-oriented interventions). This way, the LTA also suggests that improving parent–child attachment relationships requires bridging family and behavior therapy. Behavior therapists may benefit from being more attachment-emotion-focused and relational therapists may benefit from being more mindful of classical and operant learning processes.

### Parenting Support in Families with Young Children: Antecedent and Consequent Control Training

Previous research on the impact of parent management training on the parent–child attachment relationship suggests that addressing parenting skills may also have a positive effect on the parenting behaviors known to stimulate children’s secure attachment development (e.g., Matias et al., 2014; O’Conner et al., 2013). Preliminary work on Parent–Child Interaction Therapy (PCIT) for example shows that integrating an attachment perspective in parent management training may stimulate attachment-relevant parenting practices (Allen et al., 2014) but the impact on children’s attachment development may be limited (Kohlhoff et al., 2020; O’Conner et al., 2013; Timmer et al., 2011). From the LTA perspective, one reason might be that these interventions do not sufficiently account for the relational dynamics of insecure cycles and that they do not specifically target parental support to children’s insecure attachment needs.

At younger ages, interrupting and restructuring coercive cycles can be achieved working mainly with parents using, for example, Antecedent and Consequent Control Training (e.g., Forgatch & Patterson, 2010). Antecedent and consequent control training targets resolving coercive cycles using operant learning techniques (van der Oord & Tripp, 2020). Antecedent Control training targets the Sd that elicits the child’s misbehavior using rules, instructions, or by changing the stimuli in the context. If the Sd changes, misbehavior will be less functional and will decrease. Consequent Control training aims to reduce unwanted behavior by targeting the Sr. Praise, reward, and mild punishment results in decreases in unwanted child behavior and increases in wanted behavior. Successful antecedent and consequent control training first requires function analysis. Specifically, the clinician needs to explore and analyze the circumstances that elicit unwanted behavior (the antecedents) and the outcomes of this behavior that act as reinforcers and that render unwanted behavior functional, increasing the likelihood that this behavior will be repeated (the consequents).

Building on this function analysis, successful antecedent control training addresses parents’ disciplinary communication, parents’ ability to anticipate and plan for misbehavior, parents’ ability to structure the environment so that it elicits less misbehavior, parents’ use of prompts and cues to stimulate wanted behavior, parents’ use of distraction to avoid triggers for unwanted behavior (Van der Oord & Tripp, 2020). Successful consequent control training helps parents to no longer reward unwanted child behavior and to reward and stimulate instead wanted or neutral behavior. For this, parents learn to stop reinforcing unwanted behavior (e.g., using distraction or ignoring) and positively reinforce and praise wanted child behavior (Van der Oord & Tripp, 2020). Both antecedent and consequent control techniques help to interrupt coercive cycles and improve child behavior (Forgatch & Domenech Rodríguez, 2016).

#### Attachment-Focused Antecedent and Consequent Control Training

Attachment-focused antecedent control could consist of parents (1) paying more attention to the relational–emotional distress in the child’s context (CS/Sd), (2) providing safety through helping the child to manage threatening stimuli in their environment, and (3) enhancing the predictability of their availability. First, parents could learn to monitor the CSs/Sds that trigger emotions linked to not being wanted or loved and that are typically associated with problem behavior and behavioral escalations. An example could be a situation where a sibling gets more attention from the parents (e.g., during his birthday party). Second, parents can then help the child understand the situation, acknowledging that children typically feel jealous and ignored in such situations and that this is tough to deal with, but at the same time setting clear rules about what behavior is acceptable and what behavior is unacceptable. Third, parents can also explain to the child that during the birthday party they will be busy accommodating guests, but that during the party, the child can always ask for help and that after the party they will spend some time together. This approach is sensitive and supportive, and at the same time it decreases the likelihood that conflicts emerge.

Attachment-focused consequent control requires parents not only to stop reinforcing the unwanted child behaviors, but also to address children’s underlying attachment needs. For example, a child might be very attracted to a toy in a shop and show demanding behavior. Next to refusing to give in to the demanding behavior, the parent could also pay attention to the fact that desiring the toy and not getting it is distressing. By acknowledging that it is normal for children to feel like that (meaning that the child is not “bad”) and by providing comfort for the sadness and disappointment renders parents more responsive to children’s genuine attachment needs. Thus, children may learn that parents are a safety signal. This helps the parent to provide secure base support more often, which increases CS_parent_ − UCS_support_ contingency and facilitates secure attachment learning.

Such an approach fits with the well-known authoritative parenting style (e.g., Baumrind, 1967). On the one hand, authoritative parents enforce consistent limits and rules that are clearly articulated to children. On the other hand, they sensitively acknowledge children’s stress and attachment needs. Authoritative parenting requires parents to become aware which child behaviors signal the child’s distress and to express empathy for their child’s efforts to comply even if the child’s attempts fail. For example, in response to sibling aggression, a parent could say: “I see that you feel hurt and I understand that is hard to bear, but you know you cannot hit your brother”. This is secure base support. Specifically, the parent provides acknowledgement and support for the stress the child felt during the interaction with the brother. This elicits relief (e.g., my parent does not think I am a bad boy, −Sr−) and gives comfort (+Sr+). If parents’ alternative approach improves parent–child interactions, these new parental behaviors are reinforced by the child to become a stable part of the parenting repertoire. However, if parents struggle to provide that support, it becomes important to explore the reinforcers that maintain their initial parenting behavior and focus more on parents’ own psychopathology, or own attachment learning history. One way to help parents adapt their parenting behavior to new insights on their insecure cycle with their child is to work with exposure therapy.

### Attachment-Focused Therapy in Older Children: Exposure Therapy

At older ages, children’s increased information processing biases might render intervention strategies insufficient if they solely focus on changing parenting behavior. Due to these biases, older children might fail to pick up changes in their parents’ attempts to respond to their attachment needs. As a result, insecure cycles can endure even if parents manage to respond more sensitively to children’s underlying needs. Moreover, with passing time, parents’ processing of their child’s behavior will get more biased, making it harder to respond to their child’s attachment needs more sensitively. To bypass these information processing biases and related therapeutic blocks, it is necessary to create an emotionally intense learning experience that violates both parents’ and children’s expectations about feared outcomes (Tryon, 2005). One therapeutic strategy that could prove useful here is Exposure Therapy, a classical learning technique. This is a therapy during which patients are exposed to feared stimuli (CS). During exposure, the CS–UCS association will get disrupted by creating an inhibitory association. This occurs by abstaining from relying on avoidance behavior. This enables corrective learning experiences whereby patients learn that feared outcomes do not occur (Abramowitz et al., 2019).

Successful exposure therapy again requires a function analysis to chart the circumstances that elicit avoidance behavior and the reinforcers or function of this behavior. Function analysis needs to identify the stimuli or situations that elicit fears and negative expectations, the precise content of those fears and expectations, and the precise behavioral responses to those stimuli or situations (with the goal to avoid or escape the feared outcomes, which reinforces avoidance behavior). Once this information is collected, and the function analysis is sufficiently completed, exposure therapy will require participants to confront the fear eliciting stimuli or situations. During exposure excercises, therapists ensure the maximal activation of the fears and negative expectations by helping participants to abstain from avoidance behaviors and to rely on behaviors that further activate fear and negative expectations. The more all relevant fears and negative expectations are activated during exposure, the more such an exercise increases the likelihood that corrective learning occurs (Craske et al., 2014). Finally, to ensure generalization of exposure therapy effects, it is critical to repeat those exercises in different situations (Craske et al., 2014).

#### Attachment-Focused Exposure Therapy

The LTA points at exposure therapy as a relevant strategy to interrupt and/or repair insecure cycles because the theory implies that the confusing signals children and parents emit during insecure cycles are equivalent to avoidance behaviors driven by fear. Children fear and avoid the emotional pain linked to being ignored, misunderstood, left alone, or rejected by the parent. The fact that exposure therapy targets avoidance behaviors which underlie attachments could lead one to expect that exposure therapy is only a useful intervention for avoidant attachment-related behaviors. However, the repertoire of behaviors that serve to avoid a feared outcome is much broader than merely avoiding proximity. For exampe, ambivalent attachment behaviors such as protest and persuasion can help avoid feeling unseen (°Sr− or −Sr), or they can stimulate parents to try harder to accommodate the child (+Sr+). This may reduce children’s fear that their parents are unavailable (−Sr−). So, the broad spectrum of insecure attachment behaviors can be seen as avoidance behaviors to be targeted in exposure therapy.

Parents, in turn, fear and avoid the emotional pain linked to feelings of failure in their caregiving role. Attachment-focused exposure therapy is expected to be most effective when children and parents are both confronted with care-related interactions because those are the clinically relevant stimuli that elicit family members’ attachment-related fears and negative expectations. Although the precise form of the fear eliciting care-related interactions will be different from family to family, the LTA suggests that, at the most abstract level, these interactions consist of the ingredients of the insecure cycle. Thus, function analysis requires to clearly map the elements of the insecure cycle and then design exposure excercises that elicit children’s (distorted) attachment signaling which elicits parents’ non-supportive responses that feed into the insecure cycle. For corrective learning to occur during exposure, parents are stimulated to abstain from their typical responses and to attend to children’s underlying attachment needs in spite of the fears the interaction with their care-seeking child elicit. Children are stimulated to communicate their needs in a more direct manner in spite of their fears that their parents will not understand them.

If exposure excercises stimulate corrective attachment interactions, the ultimate challenge is to promote children’s secure base script development. We do not propose to expose the child to a negative parent until they no longer feel scared. Instead we expose the child to a newly trained, attachment promoting parent until this exposure challenges and revises the child’s internal negative expectation. We have shown that it is hard for insecurely attached children to process information that is incongruent with mistrust (Verhees et al., 2019). Specifically, we found in a set of cognitive bias modification experiments that attention and interpretation biases need to be bypassed before exposure to incongruent attachment information can change attachment-related expectations about the parent. To achieve this during an exposure session, therapists need to help parents and children to focus on the evidence of the secure base script during the session to support consolidating the corrective learning experience. Research on parental reminiscence suggests that discussing with the child what occurred during an interaction with parents is one promising avenue to help organizing support-related experiences in a secure base script like fashion (e.g., Apetroaia & Waters, 2018). So, clinically, it is important that the therapist explicitly points parents and children to the secure base-congruent elements of the interactions that unfolded during exposure excercises. Finally, to further stimulate secure base script learning, it is important to repeat the exposure exercises in different situations. This can include expanding the number of relevant topics that require parent–child interaction, or expanding the locations where exposure is practiced.

## Exploring the Clinical Utility of the LTA in Two Intervention Programs: VIPP-SD and MCAT

We will now explore the clinical utility of the LTA presenting two systematic interventions designed to improve parent–child attachment relationships. We will describe the background and clinical components for each intervention program. The first intervention program illustrates the application of attachment-focused antecedent and consequent control therapy, an intervention following operant learning techniques: Video-feedback Intervention to promote Positive Parenting and Sensitive Discipline (VIPP-SD) focused on young children (Juffer et al., 2017). VIPP-SD was developed some 3 decades ago before we formulated the LTA. However, the intervention was designed with the same goal to bridge the gap between attachment theory and social learning theory (Forgatch & Patterson, 2010; Juffer et al., 2017). Extensive research with randomized controlled trials points at positive effects on both attachment-relevant parenting behavior and on children’s secure attachment development (Juffer et al., 2017; Van IJzendoorn et al., in preparation). In this case, we will explore whether the probable mechanisms of change can be understood from the LTA model.

The second intervention program illustrates the application of attachment-focused exposure therapy, an intervention following classical learning techniques: Middle Childhood Attachment-based Family Therapy (MCAT; Van Vlierberghe & Bosmans, 2020). This intervention has been developed recently, explicitly building on the LTA’s insights. MCAT is developed because of the lack of systematic attachment-focused interventions that target attachment in 6–12 year old children. The intervention was designed following the logic of Attachment-based Family Therapy (ABFT; Diamond et al., 2014) an effective intervention aimed at treating depressed and suicidal adolescents by restoring parent–child attachment relationships (Diamond et al., 2016). This intervention program illustrates how the LTA can help to design new interventions in such a waythat they maximize the likelihood that secure attachment development gets restored and/or stimulated.

### Attachment-Focused Therapy for Young Children: VIPP-SD

VIPP-SD was designed to move the age for prevention of externalizing problems in young children significantly downward. Typically, families of young children who display signs of externalizing problems participate in prevention programs that build on social learning theory and that target parenting skills to disrupt or prevent coercive cycles (e.g., Patterson et al., 2004; WebsterStratton & Hammond, 1997). These programs prove effective with moderate effect sizes (e.g., Gardner et al., 2019; Piquero et al., 2016). VIPP-SD adds attachment theory to Patterson’s model of social learning to break coercive cycles. It was the first intervention that was developed with the explicit aim to integrate learning principles in an attachment-focused coaching program. The program supports parents struggling with the terrible twos, threes and older children with conduct problems (O’Farrelly et al., 2021). The program was developed with the aim to break coercive cycles and promote sensitive parenting to enhance parent–child attachment relationships (Euser et al., 2021). Given the overlap between coercive and insecure cycles, the program aligns with the abovementioned LTA-goal to interrupt insecure cycles as a mechanism to enhance the quality of parent–child interactions and attachment relationships. As we will demonstrate, to achieve this goal VIPP-SD relies partly on principles of antecedent and consequent control.

#### Secure Base Interactions in Early Childhood

VIPP-SD’s goal to shape parent–child secure base interactions requires a thorough understanding of how these interactions unfold in early childhood. Whereas in the first year of life infants frequently need physical contact to feel safe and secure, in the early childhood years the balance shifts in the direction of emotional availability of the parent in addition to moments of close physical contact when the child is tired or (di)stressed. Exploring the world is a central developmental task for the child, and this is not without the inclination to explore things such as the remote control, a precious china vase, or a road with busy traffic that the parent would rather not seeing explored by the child. In other words, parents need to balance support of their child’s explorative behavior with protection of the child and their own and others’ material possessions. Moreover, lovely babies turn into terrible twos and threes that are strong-willed and test the effects of their “No” in response to parental requests. In a secure relationship, toddlers will be non-compliant; they even *have to* be non-compliant to explore what they can achieve and what response will follow their ‘daring’ behavior. One can see the toddler check with the parent while doing something that they know they are not supposed to do. In addition, their limited behavioral control and executive functioning abilities account for little success in inhibiting themselves when doing something that they know is prohibited.

In the secure relationship, parents are aware of the children’s developing independence, and do not feel rejected by the child. They know that their child’s challenging behavior is not challenging them or their relationship. They know that it rather follows from the child’s innate drive to explore the world, and that the child will master specific skills after many trial-and-errors. These parents choose their battles, and know when to stay their grounds and when to turn a blind eye. They are consistent once they have asked a child to do something or to refrain from doing something, while showing empathy for the child when it is struggling to comply, and complimenting whatever little effort in the right direction is shown. Doing so, parents let the child know that its efforts are seen and appreciated, and that the relationship is valued.

#### VIPP-SD: Description of the Program

VIPP-SD is a six sessions program (see Table 3) that consists of two integrated components focused on stimulating secure base interactions. The attachment component is related to one of the basic goals of the VIPP-SD which is to stimulate parents’ sensitive responsiveness to the child’s signals of (di)stress and the need for physical or mental proximity to cope with distressing or threatening situations. According to attachment theory, the core of sensitive responsiveness is the parent’s ability to see the child’s attachment signals, to interpret them accurately, and to respond promptly and effectively to these signals (Ainsworth et al., 1978). Social learning is central to the second component of VIPP-SD which is to promote sensitive discipline or limit setting when the child tends to misbehave. Attachment-based interventions have focused mostly on the ‘sensitiviy’ dimension of parenting, neglecting the limit setting or discipline dimension and thus leaving parents with empty hands for example when they are faced with a toddler throwing a tantrum to have its way.

**Table 3:** Overview of the VIPP-SD program

	Sensitivity	Discipline
Session 1	Attachment and exploration	Distracting and understanding
Session 2	“Speaking for the child”	Positive Reinforcement
Session 3	“Sensitivity chain”	Sensitive Pause
Session 4	Sharing of emotions	Induction and understanding
Sessions 5 and 6 are booster sessions (repeating all themes)		

The core of sensitive discipline is preventing the occurrence of coercive cycles or interrupting these cycles when they have started. As an example, coercive cycles may start with the child wants a sweet soft drink in the supermarket, and the parent refuses to buy one because it is almost dinner time. The child throws a fit in the public space, and after a while the parent gives in, out of despair or shame. VIPP-SD aims to help parents look beyond those distorted signaling of attachment needs, set sensitive limits and at the same time provide the needed support for children’s actually experienced attachment needs. During VIPP-SD, parents learn to no longer give in, the typical and powerful immediate reinforcer of children’s unwanted behavior. Instead, they learn to approach the child’s misbehavior in such a way that it is either prevented or no longer immediately rewarded, while recognizing the child’s wishes and emotions. At the same time, parents learn to shape wanted behavior through sensitive support as a reinforcer that has both immediate rewarding effects (decrease of distress and increase of feeling connected) and long-term rewarding effects (building a more secure attachment relationship). This way, VIPP-SD fosters corrective learning experiences and the increase of CS_parent_ − UCS_support_ contingency. During VIPP-SD children learn to trust in parental support (CR) thanks to which they can start develop new behaviors to seek for parental support (R).

Essential for VIPP-SD is the use of video-feedback. Parents are not instructed in how to prevent or break a coercive cycle, or how to interact with a distressed or oppositional child. In fact, VIPP-SD uses video-taped clips of home-made, carefully selected real-life interactions between parent and child to induce a learning process at the parental level based on positive reinforcement. The emphasis in selecting video-taped fragments is on interaction sequences that were at least partially successful. Parents watch their own steps toward adequate limit setting at some (perhaps rare) occasion and see how effective their behavior can be. They experience their own ability to set limits and at the same time watch the child accepting the limits and continuing to interact with them in a trusting way.

Each of the six sessions (see Table 3) takes about one hour. Sessions are usually conducted at home although due to COVID-19 an online version has been developed as well. Video-taped interactions of the previous session are carefully studied by the coach and for each theme pertinent fragments are selected. The program is protocolized with detailed guidelines for the coaches how to proceed through the six sessions. In two-parent families the primary caregiver is present in the first four sessions, enabling the establishment of a trusting relationship between the parent and the coach. Both parents can be present in the two booster sessions.

#### LTA-Related Components of VIPP-SD: Attachment-Focused Antecedent and Consequent Control

To achieve antecedent and consequent control on insecure cycles, VIPP-SD identifies coercive cycles in the interaction between parents and their child and enhances parental strategies to prevent the occurrence of coercive cycles or to break coercive cycles at the earliest stages. In keeping with the social learning perspective, interrupting a coercive cycle means that the immediate reinforcers (see Table 2) are either removed (−Sr+) or avoided (°Sr+). This is typically achieved by having parents gently but firmly reject the child’s demand, offering an alternative or explaining why the demand cannot be fulfilled, keeping the communication open. This acts as a reinforcer of the child ending the demanding behavior (+Sr+) and shapes new interaction patterns between parents and children. VIPP-SD integrates these typical strategies with an attachment-focused approach to the coercive cycle. Specifically, VIPP-SD adds three attachment-focused elements to regular antecedent and consequent control training. These elements relate to discipline strategies, the parents’ support of children’s attachment needs, and the therapist’s positive approach to the parent, modeling the desired parent–child interactions.

First, concerning the strategies needed to interrupt coercive cycles, VIPP-SD promotes sensitive discipline to protect the parent–child attachment relationship from further ruptures. This addresses the immediate reinforcers that maintain coercive cycles, but they also ensure that parents can remain sensitive to their children’s underlying (attachment) needs. The child stays engaged with the parents and repair of the momentary rupture in the relationship is pursued (+Sr+). If achieved, this serves as a positive reinforcement for the attachment relationship and future acceptable limit setting.

Regarding sensitive discipline, parents learn not to just reject children’s demands, but to also provide reasons why the demand is misplaced or mistimed. The goal is to help parents use, for example, distraction as a means of replacing reinforcing but forbidden objects or activities with attractive alternatives. Regarding a sensitive pause, parents learn to keep its duration short and to stay in visual contact with the child. It serves the function of downregulating stress levels more of the parents than of the children. Induction is providing reasons for the limits set by the parent, making clear that the misbehaving child remains considered a valued interaction partner with the attachment relationship intact (+Sr+). Moreover, parents are encouraged to compliment the child for any tiny effort they show to comply with a parental demand. All this maximizes parent–child engagement and allows repair of momentary ruptures in the relationship resulting from limit setting. This serves as a positive reinforcement for the attachment relationship and promotes future adequate authoritative limit setting. The child’s response to sensitive limit setting is reinforcing the parents’ consistent approach to the child’s challenging behavior.

Second, to promote parents’ authoritative support of children’s attachment needs, VIPP-SD helps parents to become aware of the child’s sometimes subtle signals, and respond to them promptly and adequately. With young children, parents learn to speak for the child, to let the child know that they are seen and heard, even if a wish of the child cannot be immediately fulfilled. The parent is encouraged to share the child’s positive emotions as a way of compensating for more difficult moments. Lastly, the coach highlights so-called chains of sensitivity. Such chains of sensitivity can be made visible in the videotaped interaction when a parent responds adequately to a child signal and the child shows it is satisfied or happy with that response. For instance, this happens when the child reaches for something it is not allowed to have, the parent comments: “I see that you want another cookie but you cannot have that now, but I can make you a fruit salad if you are hungry?” and the child complies with the alternative and happily follows the mother to the kitchen. This awareness of child signals and chains of sensitivity contribute to preventing coercive cycles and ensures that positive attachment-related interactions remain a more powerful immediate reinforcer than the stimuli children gain by their demanding behavior. This way, the vicious coercive cycles are transformed into rewarding spirals of sustained interactions.

Third, to prevent the onset of insecure cycles between the coach and the parents, the coach does not act as a teacher who knows better. Instead, the coach enables the parents to explore their interactions with the child and experience the reinforcing effects of some of their more successful reactions to misbehavior of the child. In the first two sessions only positive interaction sequences are emphasized and the parents are affirmed in their role as having expert knowledge of their own child. The coach always maintains a non-judgmental attitude and acts as an empathic guide for the parents to reflect on specific rewarding parts of the videotape. Through video-feedback, already available parental skills that serve VIPP-SD’s goals are identified, shared with parents, and amplified so they can be used more in future interactions that threaten to elicit coercive cycles or in future ongoing coercive cycles.

VIPP-SD considers the role of the coach of crucial importance because the interactions and relationships of the coach with the parents function as a model for the way in which the parents interact with their child. In fact, the operant component of the LTA is relevant here. The parents feel stressed because of their problems with the child and the presence of a potentially threatening and punitive stranger who is perceived as expert and witness of their failed child rearing. It is critical in the first session to establish a non-evaluative rapport with the parents and create an atmosphere of open communication. The coach strives for empathy and sensitive responsiveness toward the parents similar to what (s)he wants to attain in the parent–child relationship. This sensitive coaching becomes then a safety cue and the discriminative stimulus on which parents can rely to seek the coach’s help and support (R) while trying to learn from VIPP-SD. The coach subsequently supports the parent while learning to break coercive circles to the point where parents feel that stress lowers (−Sr−) and state trust increases (+Sr+).

### Middle Childhood Attachment-Based Family Therapy: MCAT

Middle childhood has been long neglected in (attachment) research, and the development of attachment-focused interventions in this age-group is equally lagging behind (Bosmans & Kerns, 2015). Although VIPP-SD can be used in middle childhood as well (Runze et al., in prep.), the LTA suggests that children need to play a more active role in attachment-focused interventions as they grow older. With the LTA as basis, MCAT was developed as a transdiagnostic treatment targeting both parents and children. MCAT aims at promoting children’s secure attachment relationship with their parents to prevent further exacerbation of their emotional and behavioral problems and protect them against the impact of future (adolescence-related) stressors.

MCAT combines therapeutic elements from family therapy and behavior therapy. MCAT uses exposure therapy techniques to shape new attachment behavior in both parents and children. To facilitate attachment-focused exposure therapy in families, it is important to work with the family specific relational dynamics that drive parent–child interactions. This requires a family systems therapy approach (e.g., Bateson, 1979; Haley, 1962; Minuchin, 2018; Satir et al., 1988) as already pioneered by Bowlby (1949). Several family based attachment therapy models have been described in the literature such as Emotion-Focused Family Therapy (Lafrance et al., 2020) and Emotion Focused Family Therapy (Furrow & Palmer, 2019). However, MCAT was designed building on the ABFT model because of its strong evidence base (Diamond et al., 2016) and its explicit focus on restoring and restructuring attachment relationships. MCAT uses attachment-focused exposure therapy to restore attachment relationships and to help restructure insecure cycles. In what follows, we will mainly focus on the MCAT attachment-focused exposure therapy components.

#### Middle Childhood Secure Base Interactions

When shaping and restoring parent–child attachment relationships in middle childhood, it is important to take into account that the interactions that promote secure attachment development change hand in hand with children’s increasing maturation (Bosmans & Kerns, 2015). Research has shown that middle childhood secure base scripts are still under development and change or get updated driven by experiences linked to daily hassles (Waters, Facompre, et al., 2019; Waters, Facompré, et al., 2019). For care-related interactions to be experienced as helpful during middle childhood, the care scenario looks slightly different compared to previous developmental stages. On the one hand, middle childhood comes with novel or challenging developmental tasks that activate the attachment system and the need for care (Vandevivere et al., 2015). First, acquiring new academic skills becomes a challenge. For example, acquiring math or reading skills can be distressing and may elicit a need for parental support (Bosmans & De Smedt, 2015). Second, social interactions become a more explicit source of distress. Children struggle, for example, with peer conflicts and challenging playground interactions (Vandevivere et al., 2015).

On the other hand, there is a shift in the kind of parenting behaviors that children experience as supportive. Specifically, the supervisor role that parents already take on in certain occasions during early childhood further evolves into a *a supervisor partnership*. Parents now also help the child to explore and understand their daily hassles and their associated feelings and needs. Children no longer desire their parent to take over, instead they want their parents to help them gain insight in the issues they are faced with. Further, they find it helpful if parents propose possible solutions, allowing them to autonomously apply or improve the suggested solutions (Koehn & Kerns, 2016). However, because many of these problems and daily hassles are difficult to solve by themselves, children’s solution oriented attempts are not always successful and this activates feelings of failure in the child. At those times, it is important for children to experience *supported failure*. This refers to parents’ ability to comfort the child and show that they understand that the child did what it could do and that they do not think about the child as a failing person (Bosmans, 2016).

#### MCAT: Description of the Program

**Fig. 5:**
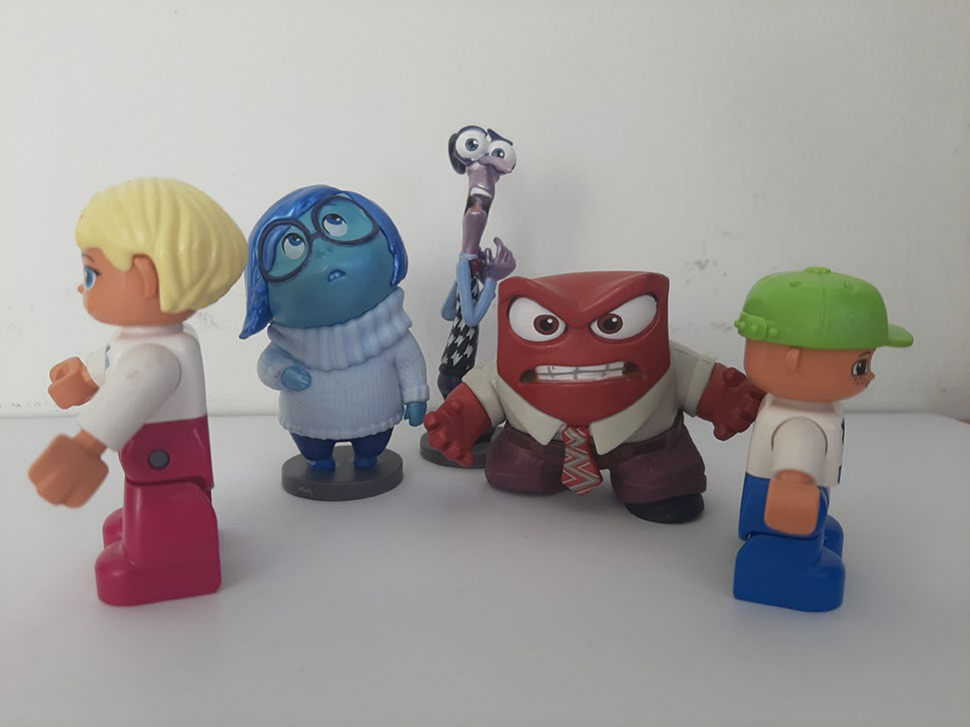
visualized family relationships and emotions

MCAT was designed accounting for the level of maturation that characterizes middle childhood. This is clearly different from ABFT, due to which it was impossible to merely apply ABFT to the middle childhood population. The fact that this is a challenging age for children to participate in family therapy is illustrated by many family therapists’ hesitation to involve children in family treatment sessions (Klop, 2017; Rober, 2008). To account for these challenges, MCAT acknowledges that younger children have not yet developed the verbal skills to fully participate in complex conversations that are part of ABFT. Instead, therapy in middle childhood requires more play and action (Klop, 2017; Rober, 2008). Therefore, MCAT is built around activities that serve as a leverage to facilitate exchange about attachment needs, emotions and unfolding interactions. For example, Lego Duplo® dolls are used to let parents and children visualize their family relationships (Diekmann-Schoemaker & van der Veer, 2003) and the emotion figures from Disney’s® movie Inside Out are used to visualize and deepen the emotional layers that drive the child’s and the parents’ behavior during insecure cycles (see Fig. 5 for an example). MCAT makes use of mental imagery exercises and various types of games, and creative techniques.

Second, MCAT builds on the observation that most children at this age have not yet acquired abstract thinking skills (Demetriou et al., 2014). These skills are necessary for ABFT, but in middle childhood, children have not yet integrated the negative experiences with their parents into abstract (insecure) attachment narratives which they can share with parents so parents can help rebuild trust. They more concretely experience and describe interactions with their parents as positive (e.g., ‘fun’, ‘nice’, or ‘good’) or negative (e.g., ‘not fun’, ‘not nice’, or ‘not helpful’). However, when currently experiencing a specific situation and when probed in a developmentally appropriate way, most children this age are able to talk more extensively and detailed about their in-the-moment thoughts, feelings and behavior, and even make links with previous situations that felt alike*.* Therefore, in MCAT, the therapist selects activities that elicit care-related interactions, which activates the child’s care script and then allows for a discussion of the child’s in-the-moment appraisals, expectations and fears regarding this type of interactions with their parents.”

Third, at this age, children’s loyalty to and dependency of their parents make it hard for them to share more vulnerable feelings and appraisals about themselves and about their parents with strangers (like the therapist in this type of brief interventions). Adolescents need child-alone sessions to feel the freedom to share their frustrations about their parents like is organized in ABFT. Instead, MCAT builds on the assumption that understanding the child side of the insecure cycle will occur more easily in the company of parents, but only after parents are prepared to listen openly to the child’s experiences. Therefore, MCAT limits the number of child-alone sessions.

In 16 one to one and a half hour sessions, organized across four phases (see Table 4) MCAT aims to (1) understand the key elements of the family’s insecure cycles (Phases 1–3); (2) motivate the family members to participate to the exposure sessions (Phases 2–4); (3) organize exposure sessions and consolidate novel learning experiences in children’s secure base script development (Phases 3–4).

**Table 4:** Overview of the MCAT program

Therapy phase	Session	Goals
1. Relational reframe	1	Switch the focus from the child as problem towards strengthening or repairing the parent–child relationship to reduce both the child’s and the parent’s suffering
2. Alliance building	2–6	(1) Build a strong working alliance with the parents and the child (2) Initiate the identification of the family specific insecure cycle
3. Interrupting the insecure cycle	7–9	(1) Start the communication about the child’s sources of distress and needs for parental support, (2) Further unravel the hypothesized insecure cycle as identified in Phase 2, (3) Empower the parents in staying out of insecure cycles with their child by (4) strengthening parents’ already available skills to provide secure base support to their child when distressed (5) and train parents’ emotion coaching skills
4. Creating secure base learning experiences	10–16	Create corrective attachment learning experiences and consolidate secure base script development

#### MCAT and Exposure Therapy

**Fig. 6:**
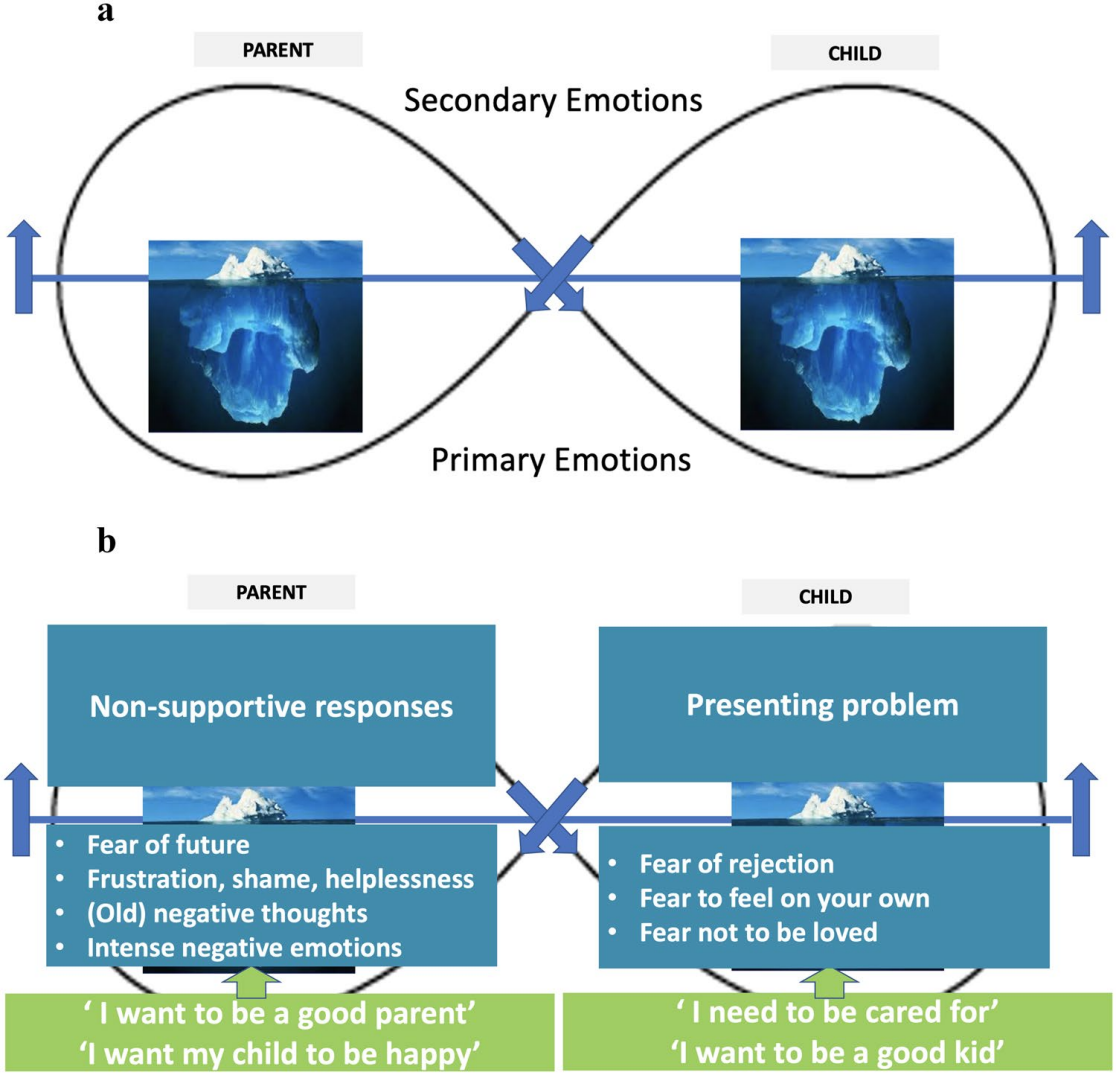
Function analysis of the Insecure Cycle

Three elements of exposure therapy are embedded in MCAT: function analysis, exposure excercises, generalization sessions. For function analysis, MCAT identifies parents’ and children’s insecure attachment-related expectations and their defensive strategies(avoidance behaviors: the child’s distorted attachment signals and the parents’ unsupportive responses) that are set in motion or perpetuate insecure cycles. To visualize this information for participating families, MCAT adjusted the infinity sign drawing developed in Emotionally Focused Therapy (Johnson, 2020; see Fig. 6). Figure 6a shows the basis of the figure. It shows the infinity sign, symbol of the ongoing interaction between parent and child. The upper side of the infinity sign (above the blue line), reflects the observable behavior and secondary emotions (e.g., anger and frustration) of both the parents and the child. In conversations with the parents on this topic, the metaphor of an iceberg is used: the upper side of the infinity sign is the part of the interaction that occurs above the waterline and that is visible. The lower side of the infinity sign (the blue line) reflects the invisible primary and vulnerable attachment-related emotions: the attachment needs and fears of both the parents and the child. The child’s primary emotions activate avoidance behavior (above the blue line), which activates the parents’ primary emotions to which they in turn respond with their own avoidance behavior. This again activates children’s primary emotions (and so on). The information gathered during Phases 1–3 serves to fill in the drawing for the family (see Fig. 6b). This drawing is used throughout the therapy to provide insight to the parents about the therapy targets. The drawing gets constantly updated if new information emerges during Phase 4 sessions.

This drawing is mainly used as a metaphor to help families understand their ongoing interactions and to look together with the therapist for alternative behavior that helps interrupt the insecure cycle. However, at the learning theoretical level, the drawing reflects past classical and operant learning processes. Specifically, the child and the child’s expression of secondary emotions are a CS for the parents (CS_parent_), which activates their insecure schemas of rejection by the child, that they will fail as a parent or that they will be considered inadequate (UCS_parent_), and the fear associated with those schemas (CR/UCR_parent_). This situation functions as a discriminative stimulus (Sd_parent_) and elicits parental avoidance behavior (e.g., aimed at convincing the child that it must not or should not feel distressed or aimed at stopping the child’s unwanted behavior; R_parent_), that gets reinforced by preventing those feared outcomes and associated negative emotions (e.g., the child stops sending signals that elicit the feeling of rejection, −Sr−_parent_, in the hope that the child will never express those signals again, °Sr−_parent_). However, as discussed in the coercive cycle section, when parents let children cross their boundaries like this, they will also feel anger and frustration about their child which more likely translates in criticism, and explicit or implicit rejection. This Sd_child_ comes with fear that they risk losing the parents’ love, which again elicits children’s missignals (R_child_) that get reinforced because they shift the focus away from feelings of loneliness and abandonment (−Sr−_child_), because they prevent those feelings to be activated (°Sr−_child_), or because they elicit parental attention (+Sr+_child_). Thus, this behavior can increase the child’s hope that parents might respond more sensitively after this new attempt (+Sr+_child_). However, this in turn becomes a Sd for the parents due to which the insecure cycle continues.

To understand the parents’ Sd (including the CR or feared emotions elicited by their child’s behavior) that activate their avoidance behavior (R_parent_), the MCAT therapist discusses with the parents the current stressors (including their worsening relationship with the child) in their lives and how they get in the way of being the parent they desire to be for the child. This way, the therapist acknowledges the parents’ good intentions and their love for the child, which helps to forge a constructive therapist-parent alliance due to which the parent can experience the therapist as a sensitive ad hoc figure and a model of a sensitive caregiver. Subsequently, the therapist explores transgenerational attachment themes, because these themes feed into the parents’ primary emotions elicited by the child’s unwanted behavior. This additionally helps the therapist to identify parents’ motivation to give their child a better childhood than what they themselves experienced as a child (if their past attachment experiences reflect substantial insecurity) or to give their child the quality of care they themselves experienced during their own childhood (if they experienced substantial security as a child).

To understand children’s Sd (including the CR or feared emotions elicited by their parents’ behavior), the MCAT therapist asks the family members to engage in activities and play that elicit attachment-relevant interaction and discourse. For example, in a blindfold game, children are asked to guide blindfolded parents through the therapist room (and vice versa). These activities typically elicit the child’s care-related script and the child behavior (R_child_) that activates the insecure cycle. Afterwards, experiences during these games that reflect confidence and uncertainty are discussed giving children ample space to discuss their attachment-related appraisals and defensive responses related to anxiety and avoidance. Hereby, the therapist works constantly on deepening emotions by activating all vulnerable emotions such that the insecure cycle is experienced by all family members during the function analysis sessions. These emotions are subsequently used and maximally activated during the exposure exercises. The exposure excercises need to expose parents to the relevant Sd_parent_ that activate past painful experiences during which they felt that children (CS) rejected them or that they failed as a parent (US_parent_) and that activate the fear that this experience will be repeated (CR_parent_). At the same time, these exercises need to expose children to the relevant Sd_child_ that have been linked in the past to the painful experience that the parents (CS) did not support or rejected them (US_hild_) and that activate the fear that this experience will be repeated (CR_hild_).

One exposure strategy is to let parents and children discuss topics that are of emotional relevance for children, such as (current) stressors (e.g., academic concerns or social problems) or family related stressors (e.g., past conflicts, frictions, or dissappointments children experienced during interactions with parents). The therapist guides the conversation between parents and children to the content that seems emotionally most relevant to the child. Such discussions will activate the child’s fear that parents will not acknowledge or understand their needs and will not provide emotional support (CR_child_). Discussing such topics will also be threatening for parents as the child might react with distorted signaling of needs, might say that have felt alone, frustrated, unheard, or misunderstood by the parents, or might tell things that trigger intense parental concerns about the child’s future development. All these responses will activate the parents’ fear to feel an unloved, incompetent, or bad parent (CR_parent_). If, during such exposure exercises, parents manage to let go of the defensive strategies (R_parent_) they typically use to protect themselves against those fears, their anxiety will increase, but it will also create the possibility for new learning experiences. Specifically, if parents manage to respond in a supportive way (distress decreases, −Sr−_child_, secure states increase, +Sr+_child_), this contradicts the child’s expectations which can result in a corrective learning experience for the child. It increases the likelihood that children let go of their self-defensive strategies (R_child_), thanks to which parents can experience that these interactions do not result in the feared rejection (−Sr−_parent_), that they get back into touch with the child (+Sr+_parent_) and that they are still loved by the child. Thus, these exposure excercises allow to disrupt the CS–UCS association due to which new experiences and expectations can be learned which facilitates relying on the desired behavior (for both the parents and the child).

Thus, the biggest challenge of attachment-focused exposure therapy in middle childhood is to help parents abstain from their typical avoidance behaviors. To achieve this goal, the MCAT therapist forges a strong collaborative therapist alliance with the parent by acknowledging their efforts, their love for their child, and (often) their desire to compensate for the care they missed as a child. Moreover, the therapist helps parents to use emotion coaching skills to replace their typical avoidant reactions. Some parents tend to reassure their children when they express distress. They try to ignore children’s distress, or they try to convince them that there is no need to feel distressed, that they should be grateful for what they have, or that they should focus on the positive. Other parents tend to emphasize how their child should try better or try to solve the problem for the child. These are only some examples of parental responses that suppress children’s attempts to share their distress. In MCAT, the therapist does not discuss with parents which of their responses are helpful or not. Instead, the therapist explains how parents can employ emotion coaching skills that facilitate a secure base discourse with their child. This means that parents do not immediately give their opinion or do not immediately try to solve the distress in response to what the child is sharing. Instead, MCAT helps parents to deepen the narrative of the child by asking questions like “can you tell me more about that” and by trying to help the child label negative emotions. This approach is taken from ABFT. Using video feedback, the therapist shows parents fragments of past sessions during which they already employ these emotion coaching skills intuitively and during which their child responds positively to open, emotion-focused discourses about the child’s distress. This strengthens parents’ sense of competence and self-efficacy and enhances the likelihood that parents respond more sensitively to the child during the exposure sessions (e.g., Mouton & Roskam, 2015).

Being asked to employ these emotion coaching skills while conversating with the child during the exposure session activates parents’ fears and negative primary emotions. Once these fears and negative emotions are maximally activated, the therapist subsequently helps the parents to use emotion coaching to prompt for children’s underlying need for care. This need contradicts parents’ fear that the child does not love them or does not appreciate them as caregivers. This is often a very emotional moment for parents on which MCAT builds to strengthen the attachment relationship. Children who feel that their parents respond in such a more emotion coaching way, more easily share their pain and fears with their parents. They seem to be in a developmental stage where their needs for care continue to drive them towards proximity seeking (see also Dujardin et al., 2019). So, if they feel that their parents are listening in an open and curious manner, without judgement, they oftentimes start sharing more suppressed negative primary emotions and cognitions. This in turn helps the parents understand their child’s distress better and often immediately softens their felt urge to employ their typical avoidance behavior. As a result the probability increases that the child feels acknowledged and supported which interrupts insecure cycles and which is a corrective attachment-related learning experience.

MCAT accounts for the major challenges these exposure sessions come along with, both for the parents and the child, by building up these sessions in increasing difficulty. Thus, the most threatening topics are touched upon once some trust has been rebuilt. Each exposure session ends when the parents are able to sustain emotion coaching until the child feels understood and supported. To consolidate the learning experience, the therapist uses “in-the-moment” verbalization (Caron et al., 2018) each time the interchange between the parents and the child reflects elements of the secure base script. To further maximize generalization of the learning experiences, the therapist ensures that parents and children practice these new communication skills about different topics. To ensure generalization, exposure occurs with the family at different locations (e.g., at the family’s home or at the child’s school during meetings with the teacher). This way, the therapist helps parents to become more atuned when children express their distress, which prevents future mistuned communication and reduces the risk that novel insecure cycles are set off. At the same time, the in-the-moment verbalisations and the generalization sessions aim to stimulate the consolidation of the learning experiences and children’s secure base script development.

## Conclusion

In this contribution, we explored the clinical applicability and utility of the LTA. We proposed that stimulating or restoring secure attachment development can occur using both operant and classical learning mechanisms. We made the point that good therapeutic strategies exist to target these learning mechanisms and discussed antecedent and consequent control training and exposure therapy as examples. We discussed how these therapeutic strategies might be adapted to fit with attachment-focused treatment targets and described two interventions that are convergent with these ideas.

We suggest that the LTA is not just promising as a theory that could inform new research on attachment development (e.g., Bosmans, Sanchez-Lopez, et al., 2019; Bosmans, Waters, et al., 2019; Verhees et al., 2021), but that the theory could also be informative for clinical practice. Obviously, much work still needs to be done into clinically relevant translations. At the level of the interventions, support for the effectivity of VIPP-SD is robust, but MCAT has only recently been developed. Moreover, for both interventions more fine-grained work on the mechanisms of change is needed. The LTA may be functional to delineate testable questions about these mechanisms.

In addition, many other clinical questions remain. For example, we now only looked at antecedent and consequent control training and exposure therapy, but these are not the only interventions that target operant and classical learning. It might be worthwhile to explore whether other interventions like, for example, cognitive restructuring can be adapted to a more attachment-focused format. However, our analysis also suggests that repairing attachment should not be seen as a mere cognitive process, but that the vulnerable emotions that drive attachment-related behaviors related to avoiding rejection and interpersonal pain should be acknowledged and taken into account.

In sum, in families with younger children, intervention programs like VIPP-SD achieve the goal of supporting parents to acknowledge the attachment needs hidden behind the child’s avoidant or resistant behaviors, and to respond in a sensitive manner. The evidence base for VIPP-SD is substantial with 25 randomized control trials in families struggling with a wide range of clinical issues showing that antecedent and consequent control training using video-feedback can raise parenting in the service of attachment security (see Van IJzendoorn et al., 2021). In older children, programs like MCAT suggest that attachment relationships can be strengthened or repaired by attachment-focused exposure therapy. MCAT is a novel program, so only limited pilot data are available, but MCAT seems to significantly increase children’s secure base script knowledge and decreases children’s presenting problems using exposure therapy.

These illustrations suggest the clinical value of the endeavor to formulate a more concrete and testable attachment theory as well as the value of clinical applications to inspire new theoretical developments. More of such cross-fertilization is needed to move this field further, to detect effective components of the programs, to integrate intergenerational attachment dynamics in a learning-based approach, to refine treatment strategies, to search for the moderators identifying the most susceptible families. We expect this interplay between LTA and clinical interventions to succeed in improving treatment outcomes, which is in the interest of the children and families that need professional support.^1^

## Supplementary Information

Below is the link to the electronic supplementary material.

Supplementary file1 (DOCX 16 KB)

## Footnotes

1 More information on training in the interventions mentioned in the current review, can be found here. For VIPP-SD: https://www.universiteitleiden.nl/en/vipp/professionals, https://www.annafreud.org/media/12018/0-5s-brochure.pdf, https://tavistockandportman.nhs.uk/training/cpd-courses/video-intervention-promote-positive-parenting-and-sensitive-discipline-vipp-sd/; For MCAT: www.learn2trust.be.
