# Supplementary material for: A Learning Theory Approach to Attachment Theory: Exploring Clinical Applications
Source: Clin Child Fam Psychol Rev. 2022 Jan 30;25(3):591–612. doi: 10.1007/s10567-021-00377-x (PMC8801239; doi:10.1007/s10567-021-00377-x)
Supplement: Supplementary file 1 — Supplementary file1 (DOCX 16 KB) [file 10567_2021_377_MOESM1_ESM.docx]

**Supplementary file 1: Young’s Schema Theory**

Based on their clinical experience, Young et al. (2003) described several types of scripts that are typically referred to as early maladaptive schemas (EMS). For example, the EMS of Emotional Deprivation reflects the expectation that one’s desire for emotional support will not be met by others, and the EMS of Defectiveness/Shame refers to the idea of being a defective, bad, unwanted, inferior, invalid or unlovable person.

In the schema-focused treatment model of Young et al. (2003) it is assumed that stressful life experiences typically activate a specific subset of EMSs in an individual, accompanied by intense negative emotions. These schemas guide how subsequent interactions with significant others are perceived, and they set off strategies to cope with or adapt to the experienced emotional pain. Young et al. (2003) refer to these states as schema modes (SMs) and describe ten types of these SMs (see Table 1). It is assumed that individuals may shift from one SM to another over time and contexts. For example, in the ‘Vulnerable Child’ mode one is surrendered to intense feelings of loneliness, abandonment, sadness, defectiveness and incompetence, while in the ‘Impulsive/Undisciplined Child’ mode one acts out of control while feeling intensively angry and frustrated. These Child modes reflect reactive states to the experience of unmet emotional needs that amplify negative emotions. In the ‘Detached Protector’ mode one detaches emotionally and rejects help from others while feeling withdrawn and disconnected and minimizes negative emotions associated with unmet needs. It is reasonable to assume that these SMs feed into the insecure cycle. They increase the likelihood that children distort attachment signals either by maximizing and amplifying their distress or by minimizing their distress and avoiding the caregiver. Parents in turn, may misread their childrens’ distorted signals and be less likely to provide comfort and support.

*Table 1 Young et al. (2003) Schema Modes*

| **Child Modes** | |
| --- | --- |
| Vulnerable Child | *Feels lonely, isolated, sad, misunderstood, unsupported, defective, deprived, overwhelmed, incompetent, doubts self, needy, helpless, hopeless, frightened, anxious, worried, victimized, worthless, unloved, unlovable, lost, directionless, fragile, weak, defeated, oppressed, powerless, left out, excluded, pessimistic* |
| Angry Child | *Feels intensely angry, enraged, infuriated, frustrated, impatient because the core emotional (or physical) needs of the vulnerable child are not being met* |
| Impulsive/Undisciplined Child | *Acts on non-core desires or impulses in a selfish or uncontrolled manner to get his or her own way and often has difficulty delaying short-term gratification; often feels intensely angry, enraged, infuriated, frustrated, impatient when these non-core desires or impulses cannot be met.; may appear “spoiled”* |
| Contented Child | *Feels loved, contented, connected, satisfied, fulfilled, protected, accepted, praised, worthwhile, nurtured, guided, understood, validated, self-confident, competent, appropriately autonomous or self-reliant, safe, resilient, strong, in control, adaptable, included, optimistic, spontaneous* |
| **Maladaptive Coping Modes** | |
| Compliant Surrenderer | *Acts in a passive, subservient, submissive, approval-seeking, or self-deprecating way around others out of fear of conflict or rejection; tolerates abuse and/or bad treatment; does not express healthy needs or desires to others; selects people or engages in other behavior that directly maintains the self-defeating schema-driven pattern* |
| Detached Protector | *Cuts off needs and feelings; detaches emotionally from people and rejects their help; feels withdrawn, spacey, distracted, disconnected, depersonalized, empty or bored; pursues distracting,  self-soothing,  or self-stimulating activities in a compulsive way or to excess; may adopt a cynical, aloof  or pessimistic stance to avoid investing in people or activities* |
| Overcompensator | *Feels and behaves in an inordinately grandiose, aggressive, dominant, competitive, arrogant, haughty, condescending, devaluing, overcontrolled, controlling, rebellious, manipulative, exploitative, attention-seeking, or status-seeking way.  These feelings or behaviors must originally have developed to compensate for or gratify unmet core needs* |
| **Maladaptive Parent Modes** | |
| Punitive Parent | *feels that oneself or others deserves punishment or blame and often acts on these feelings by being blaming, punishing, or abusive towards self (e.g., self-mutilation) or others.  This mode refers to the style with which rules are enforced rather than the nature of the rules.* |
| Demanding/Critical Parent | *feels that the “right” way to be is to be perfect or achieve at a very high level, to keep everything in order, to strive for high status, to be humble, to puts others needs before one's own or to be efficient or avoid wasting time; or the person feels that it is wrong to express feelings or to act spontaneously.  This mode refer to the nature of the internalized  high standards and strict rules, rather than the style with which these rules are enforced; these rules are not compensatory in their function.* |
| **Healthy Adult Mode** | |
| Healthy Adult | *nurtures, validates and affirms the vulnerable child mode; sets limits for the angry and impulsive child modes; promotes and supports the healthy child mode; combats and eventually replaces the maladaptive coping modes; neutralizes or moderates the maladaptive parent modes.  This mode also performs appropriate adult functions such as working, parenting, taking responsibility, and committing; pursues pleasurable adult activities such as sex; intellectual, esthetic, and cultural  interests; health maintenance; and athletic activities.* |
